# Cell Type Dependent Suppression of Inflammatory Mediators by Myocardin Related Transcription Factors

**DOI:** 10.3389/fphys.2021.732564

**Published:** 2021-10-04

**Authors:** Li Liu, Elisabeth Bankell, Catarina Rippe, Björn Morén, Karin G. Stenkula, Bengt-Olof Nilsson, Karl Swärd

**Affiliations:** ^1^Department of Experimental Medical Science, Lund, Sweden; ^2^Department of Urology, Qingyuan People’s Hospital, The Sixth Affiliated Hospital of Guangzhou Medical University, Qingyuan, China

**Keywords:** cytokines, inflammation, atherosclerosis, human coronary artery, myocardin related transcription factor, differentiation

## Abstract

Myocardin related transcription factors (MRTFs: MYOCD/myocardin, MRTF-A, and MRTF-B) play a key role in smooth muscle cell differentiation by activating contractile genes. In atherosclerosis, MRTF levels change, and most notable is a fall of MYOCD. Previous work described anti-inflammatory properties of MRTF-A and MYOCD, occurring through RelA binding, suggesting that MYOCD reduction could contribute to vascular inflammation. Recent studies have muddled this picture showing that MRTFs may show both anti- and pro-inflammatory properties, but the basis of these discrepancies remain unclear. Moreover, the impact of MRTFs on inflammatory signaling pathways in tissues relevant to human arterial disease is uncertain. The current work aimed to address these issues. RNA-sequencing after forced expression of myocardin in human coronary artery smooth muscle cells (hCASMCs) showed reduction of pro-inflammatory transcripts, including *CCL2*, *CXCL8*, *IL6*, and *IL1B*. Side-by-side comparison of MYOCD, MRTF-A, and MRTF-B in hCASMCs, showed that the anti-inflammatory impact was shared among MRTFs. Correlation analyses using human arterial transcriptomic datasets revealed negative correlations between *MYOCD*, *MRTFA*, and *SRF*, on the one hand, and the inflammatory transcripts, on the other. A pro-inflammatory drive from lipopolysaccharide, did not change the size of the suppressive effect of MRTF-A in hCASMCs on either mRNA or protein levels. To examine cell type-dependence, we compared the anti-inflammatory impact in hCASMCs, with that in human bladder SMCs, in endothelial cells, and in monocytes (THP-1 cells). Surprisingly, little anti-inflammatory activity was seen in endothelial cells and monocytes, and in bladder SMCs, MRTF-A was pro-inflammatory. *CXCL8*, *IL6*, and *IL1B* were increased by the MRTF-SRF inhibitor CCG-1423 and by MRTF-A silencing in hCASMCs, but depolymerization of actin, known to inhibit MRTF activity, had no stimulatory effect, an exception being *IL1B*. Co-immunoprecipitation supported binding of MRTF-A to RelA, supporting sequestration of this important pro-inflammatory mediator as a mechanism. Dexamethasone treatment and silencing of RelA (by 76 ± 1%) however only eliminated a fraction of the MRTF-A effect (≈25%), suggesting mechanisms beyond RelA binding. Indeed, SRF silencing suggested that MRTF-A suppression of *IL1B* and *CXCL8* depends on SRF. This work thus supports an anti-inflammatory impact of MRTF-SRF signaling in hCASMCs and in intact human arteries, but not in several other cell types.

## Introduction

There has been an intense focus on inflammation as an important driver of atherosclerosis in recent decades (Libby and Hansson, 2019), and it has been demonstrated that knockout of certain chemokines, including monocyte chemoattractant protein-1 (MCP-1 or *CCL2*), reduces atherosclerosis (Gu et al., 1998). Indeed, support for the involvement of MCP-1 (*CCL2*) in the etiology of human cardiovascular disease is strong (McDermott et al., 2005; Georgakis et al., 2019). Moreover, neutralization of the cytokine interleukin 1β (*IL1B*) was shown to reduce non-fatal myocardial infarction, non-fatal stroke, or cardiovascular death (Ridker et al., 2017), all of which are penultimate manifestations of atherosclerosis. The cellular source of the cytokines that promote atherosclerosis is uncertain, but an emerging concept is that plasticity of the resident cells of the vascular wall, including smooth muscle cells (SMCs), may allow for chemokine and cytokine release along with lipid engorgement to promote atherogenesis (Allahverdian et al., 2018; Grootaert and Bennett, 2021).

Myocardin related transcription factors (MRTFs), including myocardin (*MYOCD*), MRTF-A (*MRTFA*), and MRTF-B (*MRTFB*), act together with serum response factor (*SRF*) to drive muscle cell transcription and differentiation (Miano, 2003, 2015; Owens et al., 2004; Parmacek, 2007; Olson and Nordheim, 2010). MRTF-A and MRTF-B are regulated by actin dynamics (Miralles et al., 2003; Staus et al., 2007; Olson and Nordheim, 2010), and by stretch and matrix stiffness (Cui et al., 2015; Hadden et al., 2017), such that they become activated and translocated to the nucleus when actin is polymerized. This allows cells to adapt to mechanical cues. MYOCD shows constitutive nuclear expression, and is considered a master regulator of smooth muscle cell (SMC) differentiation (Owens et al., 2004). However, its expression level falls when SMCs undergo modulation toward a synthetic phenotype (Minami et al., 2012; Ackers-Johnson et al., 2015), and this was found to represent a causal mechanism in atherosclerosis (Ackers-Johnson et al., 2015).

Several studies have documented anti-inflammatory influences of MRTFs. Wang et al. (2012) demonstrated that bone morphogenetic protein 4 (BMP4) reduces the inflammatory mediators *IL1B*, *CXCL2*, and *CCL8* in human pulmonary artery SMCs via MRTF-A. This involves the C-terminus of MRTF-A, and suppression of a NF-κB-RelA-driven inflammation independently of SRF. Moreover, in subsequent work, it was found that MYOCD shares this anti-inflammatory property. Heterozygous MYOCD deficiency amplified surges of *IL6* and *CCL2* following stimulation of mouse aortic SMCs with interleukin 1β (Ackers-Johnson et al., 2015), and it accelerated atherosclerosis (Ackers-Johnson et al., 2015; Xia et al., 2021). MYOCD’s role as a guardian against atherosclerosis may thus depend in part on its anti-inflammatory impact in SMCs (Ackers-Johnson et al., 2015), and this may occur through RelA antagonism (Tang et al., 2008; Wang et al., 2012).

While the studies cited above support an anti-inflammatory impact of MRTFs in the vascular wall, other reports have complicated this picture. One study, using rat vascular SMCs found that MRTF-A activates *Il6*, *Il1b*, and *Ccl2* promoter activity (Yang et al., 2014), while another study found that MRTF-A aggravates lipopolysaccharide- (LPS) induced pro-inflammatory transcription in murine and human macrophages through epigenetic mechanisms (Yu et al., 2014). More recently MRTF-A was shown to enhance the angiotensin II-induced inflammatory response and aortic dissection (Ito et al., 2020; Gao et al., 2021). The basis for these apparently contradictory effects of MRTFs is unclear, but may relate to the MRTF family member studied, the cell type or species, or the pro-inflammatory stimulus. Given that atherosclerosis is such a prevalent and costly disease, it is important to clarify the cell type-dependence of the inflammatory impact of MRTFs, if all MRTFs share the same property in the same cell type, and whether the influence of MRTFs in the human coronary artery is pro- or anti-inflammatory.

## Results

### Myocardin Related Transcription Factors Share an Anti-inflammatory Impact

**FIGURE 1:**
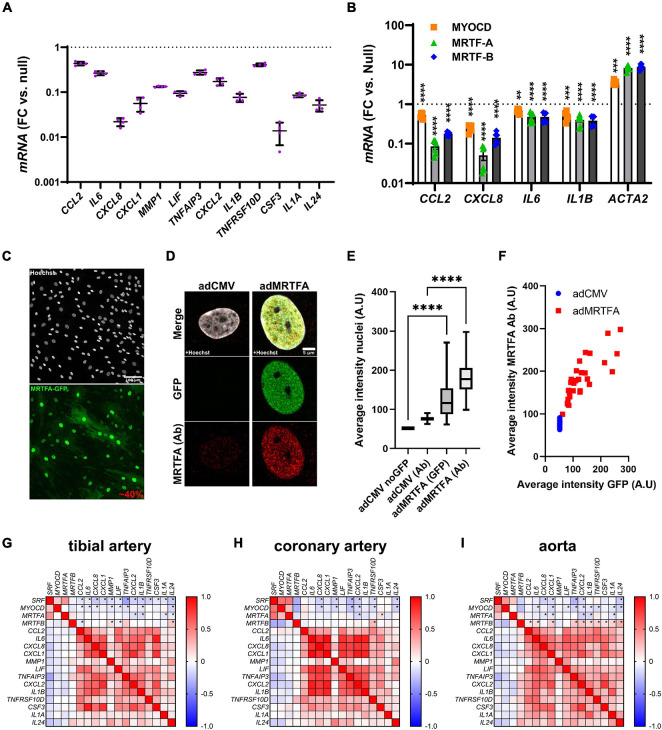
Anti-inflammatory effect of all MRTFs in human coronary artery smooth muscle cells (SMCs). **(A)** Shows mRNA levels for 13 inflammatory mediators in cultured human coronary artery smooth muscle cells (hCASMCs) after myocardin overexpression using an adenovirus (Ad-CMV-MYOCD). FC: fold change; null: Ad-CMV-null virus. Data is from an RNA-sequencing experiments conducted in a parallel study, and the control level is indicated by the dotted line. All changes were significant at an adjusted *P* < 0.0001. Suppression of inflammatory markers by myocardin (MYOCD) confirms previous studies and stimulated us to consider if this property is shared by all MRTFs. Side-by-side adenoviral transductions suggested that MTRF-A and MRTF-B have the same effect in the same cell type (hCASMCs, **(B)**). Confocal imaging showed that 40% of the cells were positive for overexpressed MRTF-A as shown using an eGFP tagged construct **(C)** and a general nuclear stain (Hoechst). In **(D)**, overexpressed and endogenous MRTF-A were labeled separately using the eGFP tag and an antibody, respectively. Quantification showed that nuclear labeling increased after viral transduction **(E)**, as expected, and the nuclear intensity of labeling in the GFP channel increased linearly with MRTF-A antibody staining **(F)**. We also examined correlations at the mRNA level in human arteries **(G–I)**. RNA-seq data was downloaded from the GTExPortal.org and correlation matrices were generated in GraphPad Prism using the Pearson method. Negative correlations (negative *R*-values, blue fills) were seen for *SRF* and *MYOCD* vs. inflammatory mediators in all arteries. Significant correlations are indicated by (*) for the first four rows in each matrix. *MRTFA* performed less well than *MYOCD* and *SRF* with only a handful significant and negative correlations in each artery. *MRTFB* performed poorly, and in this case many correlations were positive. These findings suggested a more pronounced anti-inflammatory impact of *MYOCD*, and *MRTFA* compared to *MRTFB* in the intact human vascular wall, despite similar effects upon overexpression *in vitro*. **P < 0.01, ***P < 0.001, ****P < 0.0001.

In a parallel study (submitted to the same thematic issue of this journal, Liu et al., 2021), we generated an RNA-sequencing (RNA-seq) dataset after viral overexpression of myocardin (MYOCD) in human coronary artery SMCs (hCASMCs) for 8 days. Thousands of transcripts were differentially expressed, and among them, numerous inflammatory mediators stood out as being robustly reduced (Figure 1A, adjusted *P* < 0.0001 throughout). Among the 13 transcripts plotted in Figure 1A, *CXCL8* and *CSF3* were most prominently reduced (by 97.8 ± 0.3% and by 98.6 ± 0.4%, respectively), but several were repressed by > 50%. In view of divergent findings in the literature regarding the inflammatory impact of MRTFs, we set out to examine whether repression of inflammatory mediators was a shared property among the MRTFs in the same cell type. Indeed, in side-by-side adenoviral transductions (Figure 1B), MYOCD, MRTF-A, and MRTF-B, all reduced *CCL2*, *CXCL8*, *IL6*, and *IL1B* in hCASMCs in comparison to a null construct (Ad-CMV-null or Ad-CMV). Effect sizes varied somewhat (MRTF-A > MRTF-B > MYOCD for *CCL2* and *CXCL8*), but these differences were at least partly reflected in the positive control (*ACTA2*). Taken together, these findings argue that suppression of inflammation is a shared property among the MRTFs, with only modest differences in effect between individual co-activators in the same family.

The sizeable (>90%) suppression of *CXCL8* and *CSF3* expression by MRTFs was notable because in the past we have seen that only 30–50% of the cells are positive for virally overexpressed MRTFs. We therefore examined transduction efficiency under the current experimental conditions. This was done by labeling of nuclei using Hoechst staining, and by simultaneous labeling of MRTF-A with an antibody and a GFP tag. The GFP tag should report overexpressed MRTF-A, while the antibody should mirror total MRTF-A (overexpressed and endogenous). Low magnification imaging showed that 40% of all nuclei were positive for GFP (Figure 1C, two independent experiments). Diffuse cytoplasmic staining was evident in many cells, but nuclear staining was more intense. High magnification imaging focusing on nuclei showed faint endogenous MRTF-A (antibody) staining in nuclei in control conditions (Figure 1D, left). After overexpression of MRTF-A, both GFP and antibody staining was more intense (Figure 1D, right). Quantification showed that both labels increased in nuclei after transduction (Figure 1E), and the association between nuclear GFP intensity and antibody labeling approached a linear relationship (Figure 1F). In all, these findings indicate that MRTF-A is exerting its anti-inflammatory action either inside nuclei or in the cytoplasm, and that suppression of some inflammatory mediators is greater than would be predicted from transduction efficiency (≈40%). The latter finding suggests that MRTF-A antagonizes autocrine and paracrine inflammatory feedback loops in cell culture.

### Myocardin and MRTF-A Correlate Negatively With Inflammatory Markers in Human Arteries

Our cell culture findings prompted us to examine correlations between MRTFs and inflammatory mediators in human arteries. For this, human RNA-seq data was downloaded from the GTExPortal.org and MRTFs were correlated with the inflammatory mediators identified in Figure 1A. We focused initially on the tibial artery because this dataset was the largest (*n* = 663). As expected, negative (blue fills) associations were seen when *MYOCD* was correlated with the inflammatory mediators (Figure 1G, second row in matrix). Similar analyses for *MRTFA* uncovered four negative correlations that reached the level of significance (Figure 1G, third row). Somewhat to our surprise, *SRF* performed at least as well as *MYOCD* (Figure 1G, top row), and *MRTFB* performed considerably worse than *MYOCD*, despite similar or better repression of inflammatory mediators in SMCs *in vitro* (compare Figures 1G,B). Analyses in the remaining two arteries in the database (coronary artery: *n* = 240, aorta: *n* = 432), largely echoed findings in the tibial artery (Figures 1H,I), but also emphasized that *MRTFB*, in contrast to *MYOCD* and *MRTFA*, often correlates positively with inflammatory transcripts. Taken together, these analyses suggest that MYOCD and MRTF-A, may dampen inflammation in human arteries *in situ*. We focused the remainder of this work on MRTF-A because its activity is amenable to therapy using small molecules, and because it appeared somewhat more effective *in vitro* than the other MRTFs.

### Lipopolysaccharide Does Not Affect Suppression of Inflammatory Mediators by MRTF-A

**FIGURE 2:**
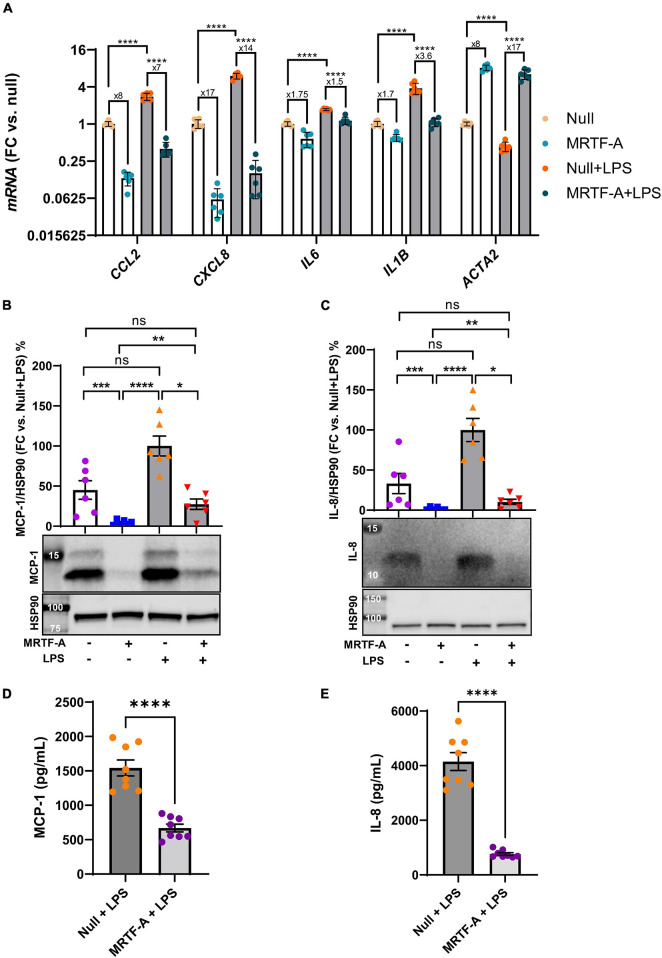
The anti-inflammatory impact of MRTF-A is maintained in pro-inflammatory conditions. **(A)** Human coronary artery smooth muscle cells in culture were transduced with Null and MRTF-A viruses in control conditions (open bars), or with simultaneous treatment with lipopolysaccharide (LPS, 500 ng/ml), to mimic a pro-inflammatory environment (gray bars). After harvesting the cells, RNA was isolated, and inflammatory transcripts were assayed using RT-qPCR. The fold repression by MRTF-A is indicated below the asterisks that indicate significance, showing that MRTF-A was similarly effective in the absence and presence of LPS. *CCL2* for example was reduced eightfold in control conditions and sevenfold in LPS treated cells, and an independent analysis indicated that this difference was not significant. To support MRTF-dependent reductions of inflammatory mediators at the protein level, MCP-1 (*CCL2*, **(B,D)**) and IL-8 (*CXCL8*, **(C,E)**) were examined using western blotting **(B,C)** and ELISAs **(D,E)**. All assays indicted significant MRTF-A-dependent reductions at the protein level, both in the absence and presence of LPS. *P < 0.05, **P < 0.01, ***P < 0.001, ****P < 0.0001.

A possible basis for discrepant effects of MRTFs on inflammation in different studies could be the inflammatory status of the cells. To address this possibility, we next compared the effect of MRTF-A in basal conditions, and under pro-inflammatory stimulation with lipopolysaccharide (LPS, 500 ng/ml), a bacterial cell wall component that activates toll-like receptor (TLR4) signaling. Using hCASMCs, all the inflammatory mediators were increased at the mRNA level by LPS. However, inflammatory suppression by MRTF-A was similar in the absence and presence of LPS. *CCL2* for example, was reduced eightfold by MRTF-A under basal conditions and sevenfold in the presence of LPS (Figure 2A, leftmost bars). Similar results were seen for the remainder of the inflammatory mediators studied (*CXCL8*, *IL6*, and *IL1B*, Figure 2A). This argued against inflammatory status as a critical factor for the direction of the effect.

Next, to ascertain that inflammatory changes at the mRNA level associate with protein level changes, we generated western blots for MCP-1 (*CCL2*), and IL-8 (*CXCL8*). MCP-1 migrated as two bands between 12 and 14 kDa, and both bands were reduced by MRTF-A in the presence and absence of LPS (Figure 2B). Similar overall suppression by MRTF-A was obtained for the IL-8 protein in western blots (Figure 2C). In keeping with the western blot results, we also found that MRTF-A reduced the levels of MCP-1 and IL-8 in cell lysates when determined using ELISAs (Figures 2D,E). MRTF-A-driven changes at the mRNA and protein levels therefore mirror each other.

### The Anti-inflammatory Impact of MRTF-A Is Cell Type Dependent

**FIGURE 3:**
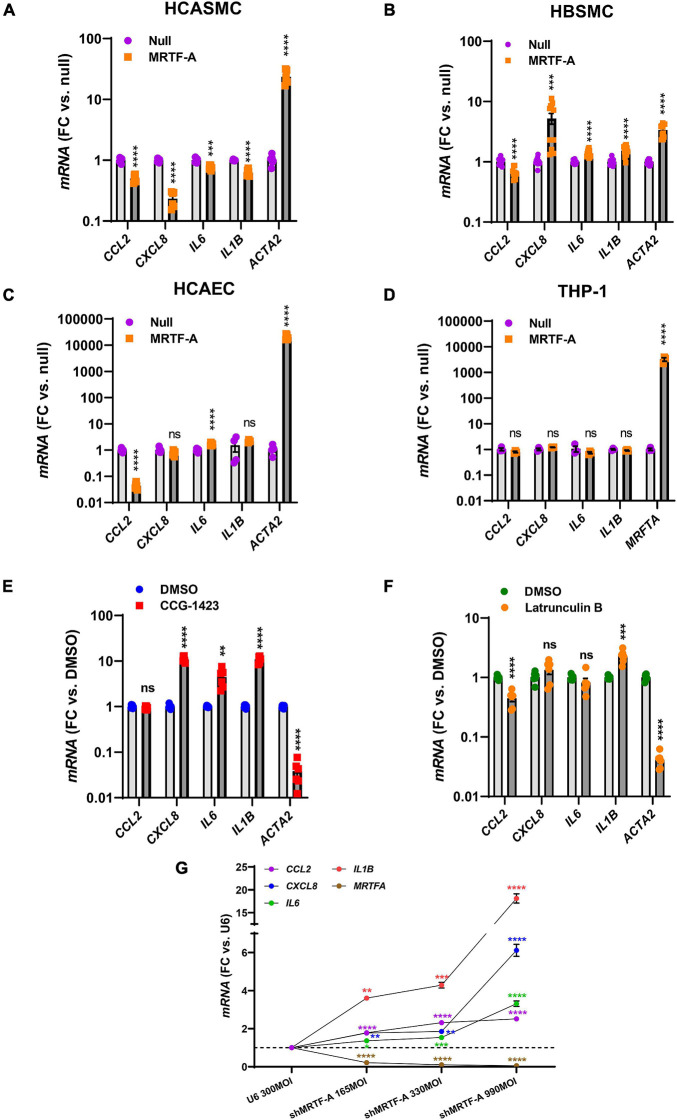
The anti-inflammatory impact of MRTF-A is cell type-dependent. To compare the impact of MRTF-A on inflammation in different cell types, MRTF-A was overexpressed using an adenoviral vector, and using a null vector as control. **(A–D)** Show the effect of MRTF-A on *CCL2*, *CXCL8*, *IL6*, and *IL1B* in human coronary artery SMCs (hCASMC, **(A)**), human bladder SMCs (hBSMC, **(B)**), human coronary artery endothelial cells (hCAEC, **(C)**), and monocytes (THP-1 cells, **(D)**). *ACTA2* was used as positive control in all panels except for THP-1 cells where we instead assayed *MRTFA* itself. **(E)** Shows the effect of the MRTF inhibitor CCG-1423 (10 μM) in hCASMC transduced with MRTF-A. **(F)** Shows the effect of Latrunculin B (100 nM), which depolymerizes actin, on the inflammatory transcripts in hCASMC. **(G)** Shows inflammatory mediators determined by RT-qPCR after silencing MRTF-A using different titers of a short hairpin virus (shMRTF-A). In this experiment U6 represents the control virus. *P < 0.05, **P < 0.01, ***P < 0.001, ****P < 0.0001.

Our findings so far showed that MRTFs share an anti-inflammatory influence, and that this effect is largely independent of inflammatory status, even if the impact of different MRTFs appears to differ considerably in the intact vascular wall. It remained possible that the cell type could matter, and it is indeed known that while MYOCD is enriched in SMCs, MRTF-A and MRTF-B are more widely expressed. We therefore next compared hCASMC with an unrelated human SMC type (bladder, hBSMC), with coronary artery endothelial cells (hCAEC), and with monocytes (THP-1 cells). Strikingly, the effects on *CXCL8*, *IL6*, and *IL1B* differed depending on cell type. Inflammatory suppression was again seen in hCASMCs as expected (Figure 3A), but in hBSMC (Figure 3B) most of the inflammatory transcripts were increased rather than decreased. In hCAEC, only two inflammatory markers changed, but in opposite directions (Figure 3C). Finally, no anti-inflammatory effect was seen in THP-1 cells even if the positive control (*MRTFA*) increased dramatically (Figure 3D). Thus, the inflammatory impact of MRTFs seems to be highly cell type dependent.

### Myocardin Related Transcription Factor Activity Can Be Manipulated to Modulate Inflammation

Small molecule inhibitors of MRTF-SRF signaling have been developed with a view to treat cancer and fibrosis. One of these is CCG-1423, and it inhibits MRTF-SRF driven gene activation with an IC_50_ value in the micromolar range (Evelyn et al., 2007). We predicted that CCG-1423 should increase the inflammatory transcripts in hCASMC if endogenous MRTFs constitutively suppress inflammation. Indeed, with exception for *CCL2*, the inflammatory transcripts were increased by CCG-1423 (10 μM) while the positive control *ACTA2* was reduced as expected (Figure 3E).

An important property of MRTF-A and MRTF-B is that they are regulated by the ratio of monomeric to polymeric actin. This depends on binding of MRTF-A and MRTF-B to monomeric actin in the cytoplasm via so called RPEL-motifs. When actin is polymerized, MRTFs move to the nucleus. We therefore treated cells with Latrunculin B (100 nM) which depolymerizes actin, expecting to see increases of the inflammatory transcripts. However, with exception for *IL1B*, none were increased, and *CCL2* was reduced (Figure 3F).

To further support an anti-inflammatory action of endogenous MRTF-A we used a short hairpin construct for silencing (shMRTF-A). In keeping with the effect of CCG-1423, silencing of MRTF-A had a significant pro-inflammatory effect with the largest effects seen for *IL1B* and *CXCL8* (Figure 3G).

### Further Mechanistic Insight

**FIGURE 4:**
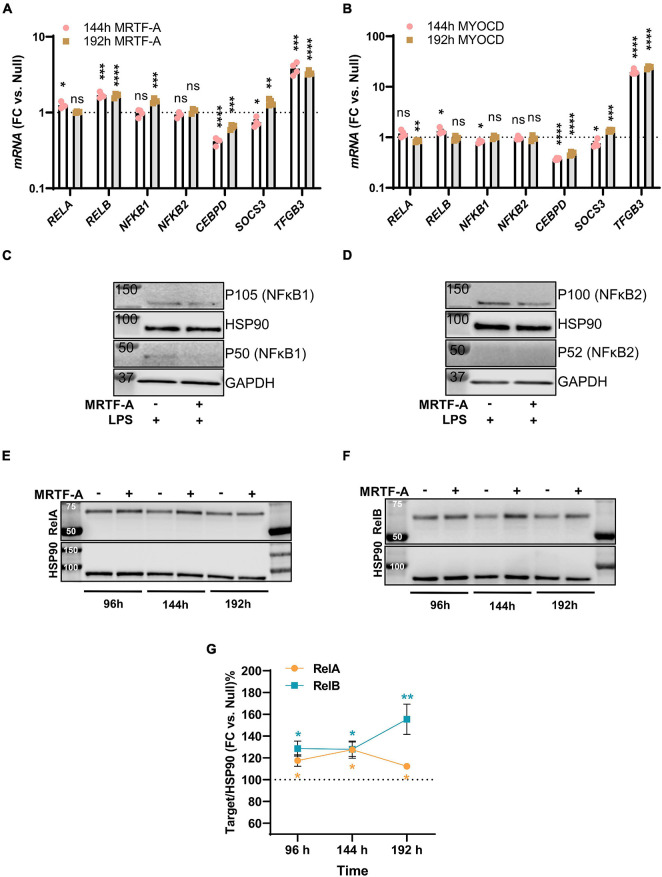
Only modest remodeling of the inflammatory apparatus by MRTF-A. Transcripts of possible relevance for inflammation were identified in an RNA-seq experiment where MYOCD was overexpressed in human coronary artery smooth muscle cells. These transcripts were then examined by RT-qPCR at two different times of overexpression of MRTF-A **(A)** and MYOCD **(B)**, respectively. **(C,D)** Show western blots after treatment with null virus and MRTF-A virus in the presence of LPS. The only consistent differences observed by western blotting were modest increases of RelA **(E,G)** and RelB **(F,G)**. *P < 0.05, **P < 0.01, ***P < 0.001, ****P < 0.0001.

Among the mechanisms that have been proposed to underlie MRTF suppression of inflammation is inhibition of NF-κB signaling through direct interaction with RelA in the nucleus. This subsequently interferes with RelA recruitment to the *IL1B* and *CXCL2* promoters (Wang et al., 2012). Another proposed mechanism is suppression of *CEBPB* and *CEBPD* (Ackers-Johnson et al., 2015), which are important for sustained inflammation. To approach these as possible mechanisms, we first surveyed our initial RNA-seq experiment with MYOCD for plausible targets that were differentially expressed and performed confirmatory RT-qPCR analyses using independent samples. Levels of *RELA*, *RELB*, *NFKB1*, *NFKB2*, *CEBPD*, *SOCS3*, and *TGFB3* are shown for MRTF-A and myocardin overexpression vs. null at two different times of transduction in Figures 4A,B, respectively. With exception for *CEBPD* and *TGFB3*, no changes were consistent for both times and both coactivators, despite inflammatory suppression by both MRTFs at both times (not shown).

We also examined some of these mediators at the protein level. No measurable changes of NFκB1, and NFκB2 were detected under basal conditions (not shown). After stimulation with LPS, P105 (NFκB1) and P100 (NFκB2) were reduced in most samples (Figures 4C,D), but this was not reflected in group averages (not shown), and the active forms (P50/P52) could not be reliably quantified. The only consistent finding at the protein level was that RelA and RelB were increased as shown in independent time-course studies (Figure 4E through 4G), and this was consistent with small increases at the mRNA level, at least for RelB (compare Figures 4A,F). Altogether, this suggested that protein level changes of NFκB1, NFκB2, RelA, or RelB are unlikely to contribute to the anti-inflammatory effect of MRTF-A. We did not further pursue *CEBPB* and *TGFB3* as mechanistic explanations, because they were reported to be important for late phase resolution of inflammation. Taken together, we thus felt that the reported direct interaction between MRTF-A and RelA (Tang et al., 2008; Wang et al., 2012), occurring independently of SRF, appeared as the most attractive mechanism.

**FIGURE 5:**
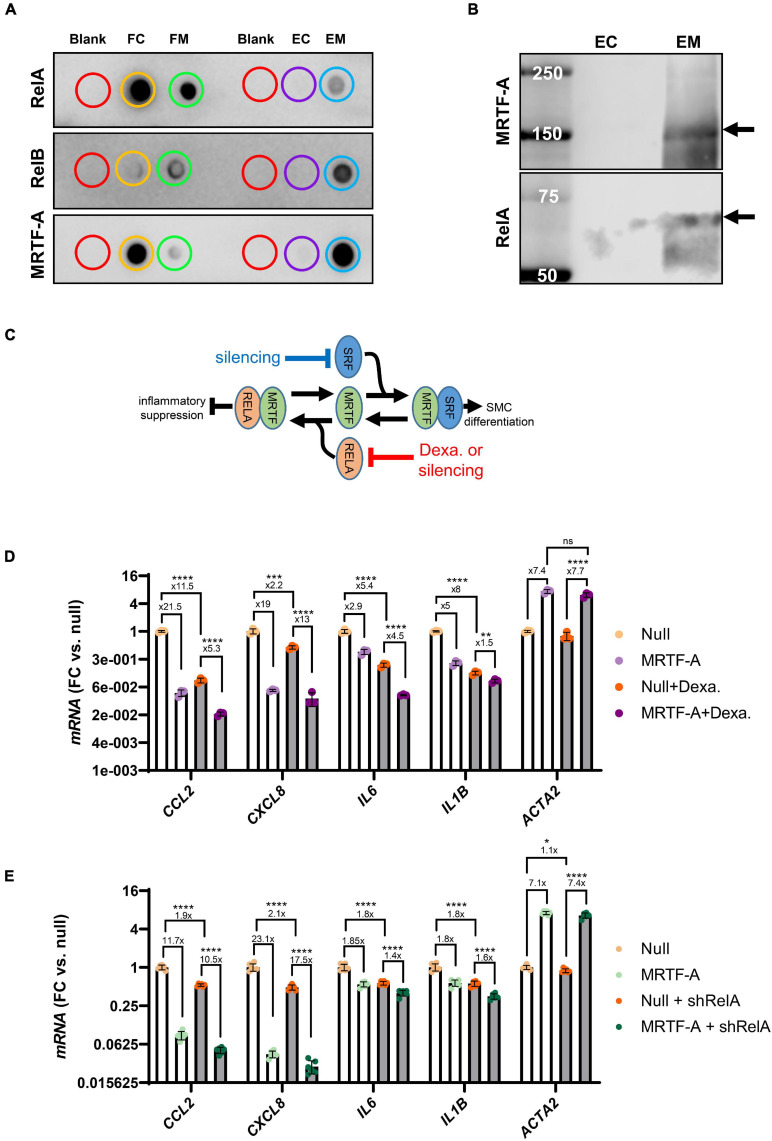
MRTF-A interacts with RelA and RelB. To explore if MRTF-A binds RelA and RelB, we performed co-immunoprecipitations followed by dot blotting **(A)**. Lysates from cells where MRTF-A was overexpressed were incubated with control resin and with MRTF-A-antibody-conjugated resin. Flow through (FC: flow through control; FM: flow through MRTF-A) from both columns contained RelA, RelB, and MRTF-A. RelA, RelB and MRTF-A were moreover detectable in the eluate from the MRTF-A column (EM: eluate MRTF-A) but in the in the eluate from the control column (EC: eluate control). Western blotting **(B)** of the eluates showed the RelA band at the expected molecular weight. These findings support a model where MRTF binding to Rel proteins suppresses inflammation **(C)**. **(C)** Also depicts the hypothesis that RelA suppression, by glucocorticoid receptor stimulation or silencing, should mitigate inflammatory suppression by MRTF-A. **(D,E)** Test this hypothesis using dexamethasone (glucocorticoid receptor agonist, 3 μM) or short hairpin silencing (200 MOI of shRELA). For the experiments in **(D,E)**, the fold repression is given. Independent testing showed that the fold repression of *CCL2* and *IL1B* by MRTF-A was reduced by dexamethasone. In contrast, fold suppression of *CXCL8* and *IL6* was unaffected. For RelA silencing, *CXCL8* and *IL6* suppression by MRTF-A were significantly reduced, but the overall effect, where MRTF-A suppression of inflammation was reduced by ≈25%, was also significant. *P < 0.05, **P < 0.01, ***P < 0.001, ****P < 0.0001.

To support interaction between MRTF-A and Rel proteins we next performed co-immunoprecipitation (co-IP) experiments. Control and MRTF-A antibody resins were incubated with lysates from cells where MRTF-A had been overexpressed. After washing and elution, dot blotting was performed (Figure 5A). RelA, RelB, and MRTF-A were detectable in the flow through (FC: flow through control; FM: flow through MRTF-A), as expected. Importantly, RelA and RelB were also detected in the eluate from the MRTF-A antibody resin (EM: MRTF-A eluate), but not in the eluate from the control resin (EC: control eluate; Figure 5A). This was particularly striking for RelB. Similar results were obtained for RelA when assayed in the eluates using western blotting (Figure 5B). Altogether, this supported the previously reported model that MRTF-A may bind and sequester Rel proteins, causing inhibition of inflammatory signaling as depicted graphically in Figure 5C.

In the inactive state, RelA and p50 are bound by Inhibitor of κB (IκBα) in the cytosol. Upon inflammatory stimulation, such as with LPS, IκBα is degraded, releasing RelA for nuclear translocation. Corticosteroids, including dexamethasone, prevent nuclear translocation of the RelA complex (Clark, 2007). We therefore predicted that inflammatory suppression by MRTF-A should be smaller in the presence of dexamethasone (as depicted graphically in Figure 5C). To test this, we overexpressed MRTF-A in control conditions and after treatment with dexamethasone (3 μM). The inflammatory mediators were then assayed by RT-qPCR. We observed that MRTF-A-driven suppression of *CCL2* and *IL1B* was smaller after treatment with dexamethasone compared to vehicle (*P* < 0.0003 for relative suppression, using Null and Null + dexamethasone independently for normalization, note that Figure 5D shows data normalized to Null only). This appears consistent with our co-IP experiment and with the work of Wang et al. (2012), showing Rel titration as the key mechanism. Importantly, relative suppression of *CXCL8* was not significantly affected (13- vs. 19-fold, *P* > 0.05), and the effect of MRTF-A on *IL6* was enhanced in the presence of dexamethasone (4.5- vs. 2.9-fold, *P* = 0.0043). This suggested that MRTF-A-RelA interaction could contribute to repression of a subset of the inflammatory mediators (*CCL2*, *IL1B*), but that other mechanisms may be involved for some of them (*CXCL8*, *IL6*).

To sharpen this conclusion, we repeated the same experiment with short hairpin silencing of RelA (by 75.6 ± 1.0%, *P* < 0.0001, *n* = 12, Figure 5E). While the trend was that the anti-inflammatory effect of MRTF-A was dampened (*CCL2*: 11.7-fold→10.5-fold, *CXCL8*: 23.1-fold→17.5-fold, *IL6*: 1.8-fold→1.4-fold, *IL1B*: 1.8-fold→1.6-fold), the overall effect was a mere 25% dampening of fold repression (overall *P*-value: 0.0043), and only the effects on *CXCL8* (*P* = 0.026) and *IL6* (*P* = 0.0019) were individually significant. Taken together, these findings argue that mechanisms beyond Rel binding and inhibition are involved in inflammatory suppression by MRTF-A.

### Serum Response Factor Is Involved in Suppression of *IL1B* and *CXCL8* by MRTF-A

**FIGURE 6:**
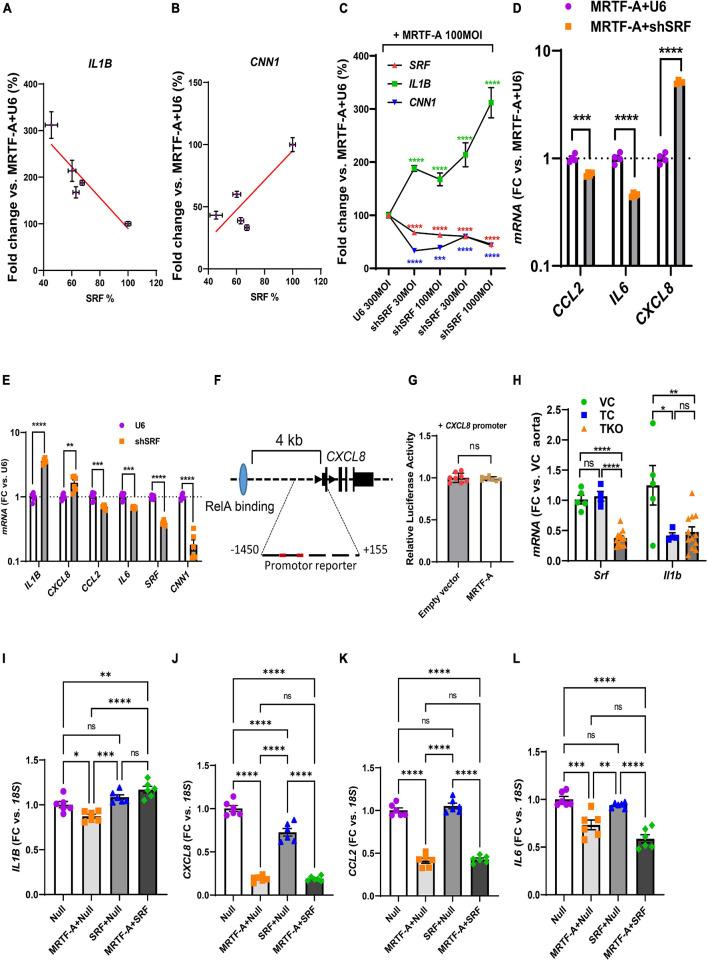
SRF is important for regulation of *IL1B* and *CXCL8*. **(A–C)** Human coronary SMCs were transduced with MRTF-A along with different titers of Ad-h-shSRF for knockdown of SRF. After harvesting the cells and isolation of RNA, *SRF*, *IL1B*, and *CNN1* were assayed using RT-qPCR. In **(A,B)**, *IL1B* and *CNN1* were plotted vs. the relative *SRF* level in the respective samples. In **(C)**, *SRF*, *IL1B*, and *CNN1* were plotted vs. the titer (multiplicity of infection, MOI) of the short hairpin virus. **(D)** Shows the remainder of the inflammatory mediators in control vs. SRF-depleted cells (1000 MOI). **(E)** Is like **(D)**, except that SRF silencing was done without simultaneous overexpression of MRTF-A. **(F)** Shows the gene locus for human *CXCL8* and binding of RelA (light blue oval) 4 kb upstream of the transcription start site. The proximal promoter contained two DNA sequences with two deviations each from the classical SRF-binding sequence. When testing this sequence in a reporter assay, no suppression by MRTF-A was however, seen **(G)**. In **(H)**, *Srf* and *Il1b* were assayed in the aorta from smooth muscle specific and inducible Srf knockout mice. Because knockout was induced by tamoxifen, two control groups were included in addition to the knockout group. Vehicle controls (VC) are Cre-positive mice injected with sunflower oil, whereas tamoxifen controls (TC) are Cre-negative mice injected with tamoxifen. Knockouts (TKO) are Cre-positive mice injected with tamoxifen. All mice are homozygous for the floxed *Srf* allele. **(I–L)** Show the effect of overexpression of SRF in the absence and presence of MRTF-A. SRF was capable of suppressing *CXCL8* on its own but was without effect on *CCL2* and *IL6*. *P < 0.05, **P < 0.01, ***P < 0.001, ****P < 0.0001.

Our findings so far highlighted mechanisms beyond RelA titration by MRTF-A for inflammatory suppression, and our correlation analyses using human arteries suggested a possible role of SRF. We therefore next examined silencing of SRF using a short hairpin construct (Ad-shSRF). We focused initially on *IL1B* in view of its relevance for cardiovascular disease and we used hCASMC where MRTF-A had been overexpressed. In this setting, gradual reduction of *SRF* using four different doses of silencer, increased *IL1B* (Figure 6A, negative slope), while the classical MRTF-SRF target gene calponin (*CNN1*) was reduced (Figure 6B, positive slope). This was also evident when data was plotted relative to the dose of the virus (multiplicity of infection, MOI) rather than relative to the level of SRF (Figure 6C). Suppression of *IL1B* by MRTF-A therefore depends on SRF in the setting of silencing.

To determine the effect of SRF silencing for the remainder of the inflammatory mediators, we used the RNA from cells treated with the highest titer of Ad-shSRF virus in Figure 6C. Beyond *IL1B*, *CXCL8* was also increased as shown in Figure 6D. *CCL2* and *IL6* on the other hand were reduced. This argues that SRF is involved in suppression of *IL1B* and *CXCL8* by MRTF-A, and that, in the SRF silencing situation, *CCL2* and *IL6* behave as if their regulation depended more on the reported (Wang et al., 2012) RelA sequestration by MRTF-A (that is, silencing of SRF makes more MRTFs available for RelA binding according to the model in Figure 5C). To corroborate the SRF dependence of *IL1B* and *CXCL8*, we next repeated SRF silencing without simultaneous overexpression of MRTF-A. Silencing of SRF again increased *IL1B* and *CXCL8* (Figure 6E) while *CCL2* and *IL6* were reduced. In all, this suggested MRTF-A-SRF signaling as a mechanism of suppression of *IL1B* and *CXCL8*, and MRTF-A dependent RelA titration as a mechanism of suppression of *CCL2* (and possibly *IL6*).

To explore the possibility that SRF acts directly via DNA elements at the *CXCL8* (IL-8) locus, we examined this sequence in the genome browser. ENCODE ChIP-seq data supported RelA binding ≈4 kb upstream of the gene (Figure 6F, blue ellipse). No SRF binding was documented. Putative SRF-binding sequences were however noted within 1450 bases from the transcription start site (red sites in Figure 6F). We therefore used this sequence in a promoter reporter assay. This promoter was however not suppressed by MRTF-A (Figure 6G), arguing that regulation of *CXCL8* by MRTF-SRF signaling depends on other DNA regions, perhaps regions in the vicinity of the more distant RelA-binding site.

We next sought to examine if Srf is important for *Il1b* expression *in vivo*. For this we used smooth muscle specific and inducible knockouts of Srf. Cre-mediated deletion of Srf was achieved by intraperitoneal injections of tamoxifen for 5 consecutive days in mice carrying floxed *Srf* alleles and a tamoxifen activatable and smooth muscle specific Cre transgene. Mice were euthanized on day 20–21 after the first injection. We used both Cre-positive vehicle injected mice (VC) and Cre-negative mice injected with tamoxifen (TC) as controls and compared these with the tamoxifen inducible knockouts (TKO). After isolation of RNA from the aorta, we measured *Srf* and *Il1b* using RT-qPCR. The two control groups did not differ with respect to Srf expression (Figure 6H, left), and *Srf* was depleted in the knockouts (TKO) as expected. However, aortic *Il1b* was reduced by tamoxifen in the control mice, and no increase from this level was apparent in the Srf knockouts (Figure 6H). This argued that tamoxifen has an anti-inflammatory effect that is independent of Srf depletion and that may hide Srf-dependent regulation. This makes the model unsuitable for studying inflammation.

We finally overexpressed SRF both alone and together with MRTF-A in hCASMCs. Curiously, repression of *IL1B* was lost on overexpression of SRF (Figure 6I). For *CXCL8*, we noted that SRF had a suppressive effect on its own (Figure 6J). For *CCL2* and *IL6* MRTF suppression remained unchanged by simultaneous overexpression of SRF (Figures 6K,L). Overexpression of SRF therefore further implicates SRF in suppression of *CXCL8*, but this experiment is difficult to interpret for the remainder of the inflammatory mediators.

## Discussion

The present work confirms previous studies showing that MRTFs have an anti-inflammatory impact in certain cell types, most notably hCASMC. However, the outcomes of this investigation are intriguing in some important and novel regards. One is that we find all MRTFs to exert an anti-inflammatory effect when overexpressed in the same primary cell type, and yet only MYOCD and MRTF-A leave a suppressive mark on inflammatory transcripts in intact human arteries. The latter finding was made using the largest material yet exploited in support of inflammatory suppression by MRTF-A/MYOCD in the vascular wall of humans. Despite this, knockout of MYOCD or MRTF-A have directionally opposite effects on atherosclerosis in the mouse. That is, while homozygous deletion of MRTF-A reduces atherosclerosis (Minami et al., 2012), hemizygous deletion of MYOCD increases it (Ackers-Johnson et al., 2015). Upon first reflection, this contradiction makes little sense, but it is possible that the different MRTFs are expressed in entirely different cell types with different roles in vascular inflammation. Indeed, in parallel work (Liu et al., 2021) we find that MYOCD is enriched in SMCs as expected, and that MRTF-B is enriched in endothelial cells, while MRTF-A primarily resides in fibroblasts. Additional explanations for the discrepant effects of MRTF-A and MYOCD in atherosclerosis include coactivator-specific effects. One such effect, that appears to differ between different MRTFs, relates to lipid uptake. While MYOCD reduces lipid uptake into hCASMCs (Ackers-Johnson et al., 2015), MRTF-A has the opposite effect as it increases low density lipoprotein (LDL) uptake via LDL receptors (Alajbegovic et al., 2021). Overall, the impact of different MRTFs on atherosclerosis therefore seems most consistent with their reported effects on lipid uptake.

On the mechanistic level, we report an important, and previously overlooked, finding, namely that SRF is somehow involved in suppression of some inflammatory transcripts by MRTF-A. This includes *IL1B* whose neutralization in a clinical trial was found to reduce cardiovascular mortality (Ridker et al., 2017), and *CXCL8*. Our findings with *CCL2*, on the other hand, are consistent with the reported RelA titration by MRTF-A (Wang et al., 2012). Therefore, not one mechanism, but many, must be responsible for MRTF-dependent suppression of inflammation. SRF is important, often critical, for binding of MRTFs to DNA, and such binding in the vicinity of the *IL1B* and *CXCL8* loci could impede attachment of another stimulatory factor. Alternatively, the effect could involve a downstream target gene, or e.g., chromatin remodeling. We were not successful in further defining the DNA regions involved using a promoter reporter assay for *CXCL8*, but our efforts were not exhaustive.

When attempting to support involvement of Srf in mice *in vivo*, we observed that tamoxifen, required for Cre-mediated gene excision in our model, had an anti-inflammatory impact 21 days after the first tamoxifen injection. We are uncertain of the basis of this anti-inflammatory effect, which could involve promiscuous binding of steroid-like chemistries to glucocorticoid receptors, or estrogen receptor modulation, but similar effects have been reported previously (Lamas et al., 2015). We are therefore forced to conclude that tamoxifen-dependent gene excision is unsuitable for studying the role of MRTF-SRF signaling in inflammation. Importantly, however, involvement of SRF for *CXCL8* regulation was demonstrated in cultured SMCs using both silencing and overexpression, and it was further supported by correlation analyses in a large human material.

It is currently unclear to us why forced overexpression of MRTF-A has so drastically different effects in different cell types. Inflammatory status is not a key underlying factor as suggested by our LPS experiments. However, some other cell-specific factor must play a role because we see rather different effects of MRTF-A in coronary and bladder SMCs. Identification of this factor could perhaps resolve the puzzling fact that MRTFs may be both pro- and anti-inflammatory. It is interesting to note that the proposed mechanism of the pro-inflammatory impact of MRTF-A in THP-1 cells involves the epigenetic modifiers Ash2, Wdr5, and Set1 (Yu et al., 2014). At least one of these (Ash2/*ASCL2*) is expressed at a very low level in the coronary artery (GTExPortal.org). One possibility therefore is that Ash2 determines the directionality of the effect.

Here, we show that overexpression of MRTFs downregulate several pro-inflammatory transcripts and proteins relevant for vascular pathophysiology and development of vascular disease. However, the RNA-seq experiment providing impetus for the present work (Liu et al., 2021) revealed additional pro-inflammatory genes, such as *SGPP2* (sphingosine 1-phosphate phosphatase 2), *IL32* (interleukin 32), and *TSLP* (thymic stromal lymphopoietin), as being repressed. Notably, downregulation of *SGPP2* gene activity and sphingosine 1-phosphate phosphatase 2 protein levels reduces TNFα-induced *IL1B* and *CXCL8* production in endothelial cells, and moreover, knockdown of NF-κB/RelA shows that SGPP2 is an NF-κB-regulated gene (Mechtcheriakova et al., 2007). Our RNA-seq experiment also revealed that *LACC1* (laccase domain containing 1), which encodes an oxidoreductase that stimulates fatty-acid oxidation, increases (3.3-fold). Loss of function mutations in *LACC1* associate with several inflammatory diseases, such as juvenile idiopathic arthritis and Crohn’s disease (Szymanski and Ombrello, 2018), offering the interesting possibility that MRTFs may antagonize inflammation in part via upregulation of *LACC1*. Yet another example is *DUSP1*, which is partly responsible for the anti-inflammatory action of dexamethasone (Abraham et al., 2006), and that was increased 1.5-fold. Thus, MRTFs may regulate many genes associated with inflammation beyond those corroborated here, underscoring a pleiotropic anti-inflammatory action, and making these coactivators possible targets for treatment of inflammatory diseases including atherosclerosis.

MRTFs, particularly MRTF-A, are sensitive to a variety of mechanical inputs, including substrate stiffness (Foster et al., 2017; Hadden et al., 2017), externally applied forces (Zhao et al., 2007; Chan et al., 2010; Cui et al., 2015), and geometric constraints (Jain et al., 2013). Blood pressure as well as arterial stiffness could therefore affect vascular inflammation via MRTFs, and this could be a way to compensate for the increased lipid uptake into SMCs via LDL receptors (Alajbegovic et al., 2021). However, mechanical signals to MRTFs involve the actin cytoskeleton (Zhao et al., 2007; Chan et al., 2010; Sward et al., 2016), and we find here that depolymerization of actin does not consistently increase the inflammatory mediators regulated by MRTF-A; in fact *CCL2* was significantly decreased, while *IL1B* increased. Our findings therefore caution against generalizations and calls for studies of distinct target genes, rather than relying on reporter assays, and comparing the impact of different mechanical modalities and protocols. It would, for example, be of interest to know if there was such a thing as a healthy level of mechanical input on SMCs in the vascular wall, akin to the local optimum of MRTF activity seen on substrates of different stiffness (Hadden et al., 2017). Nonetheless, using reporter assays, others have shown consistent increases of NF-κB activity using Latrunculin (Jain et al., 2013).

In summary, the present work confirms previous reports showing that MRTFs have a broad anti-inflammatory impact by suppressing numerous cytokines in human coronary artery SMCs. This effect is equal to, or greater, than the effect of dexamethasone. We also find that *MYOCD*, *MRTFA*, and *SRF* correlate negatively with many inflammatory transcripts in human arteries, supporting an anti-inflammatory impact *in situ*. Our mechanistic studies suggest that the underlying mechanism of action cannot solely depend on RelA sequestration, and that SRF appears to be involved in regulation of *IL1B* and *CXCL8*. Taken together, this work supports the concept that phenotypic modulation of SMCs involves toggling between contractile and inflammatory phenotypes, in addition to the classical paradigm where SMCs switch between contractile and synthetic phenotypes (Thyberg et al., 1983; Miano, 2003).

## Materials and Methods

### Primary Cell Culture, Viral Transduction, and Cell Treatments

Human coronary artery smooth muscle cells (hCASMCs, Thermo Scientific/Gibco, C0175C) were cultured in Medium 231 (Thermo Scientific, M231500) with growth supplement (5% SMGS: Life Technologies, S00725) and 50U/50 μg/ml penicillin/streptomycin (PEST, Biochrom, A2212). Human bladder smooth muscle cells (hBSMCs) were isolated from human detrusor strips (Zhu et al., 2017) and cultured in DMEM/Ham’s F-12 medium with glutamine (Biochrom, FG4815), 10% fetal bovine serum (FBS: Biochrom, S0115), and 50 U/50 μg/ml PEST. Human coronary artery endothelial cells (hCAECs, Lonza, CC-2585) were cultured in EGM-2 MV, Microvascular Endothelial Cell Growth Medium-2 BulletKit (Lonza, CC-3202), which contains EBM-2 Basal Medium (Lonza, CC-3156) and EGM-2 MV Microvascular Endothelial Cell Growth Medium SingleQuots supplements (Lonza, CC-4147). These primary cells were kept in a standard cell culture incubator at 37°C, in 95% air and 5% CO_2_, and used in passages 3–9.

Adenoviral vectors for overexpression and silencing were purchased from Vector Biolabs. Ad-h-MYOCD (ADV-216227), Ad-h-MKL1/eGFP (MRTF-A, ADV-215499), Ad-h-MKL2 (MRTF-B, ADV-215500), Ad-h-SRF (ADV-224323) and Ad-CMV-Null (#1300) were for overexpressing target genes. Ad-h-shSRF (shADV-224323), Ad-h-shRELA (shADV-220994), Ad-U6-h-MKL1-shRNA (shADV-215497) and Ad-GFP-U6-shRNA (#1122) were for silencing target genes. Among them, Ad-CMV-Null (#1300) and Ad-GFP-U6-shRNA (#1122) were used as negative controls. Cells were harvested at 96 h after viral transduction unless specified.

CCG-1423 is a Rho/SRF pathway inhibitor and it was purchased from Tocris Bioscience (#5233). After 24 h in low-serum conditions (2% SMGS), hCASMCs were treated with 10 μM CCG-1423 or the corresponding volume of DMSO (Sigma-Aldrich, #D5879) in 2% SMGS M231 medium for 24 h.

Latrunculin B for depolymerizing actin was purchased from Calbiochem (#428020). After 24 h in low-serum conditions (2% SMGS), cells were treated with 100 nM Latrunculin B or the corresponding volume of DMSO (Sigma-Aldrich, #D5879) in 2% SMGS M231 medium for 24 h. Cells were then harvested for isolating RNA.

LPS (*E. coli* LPS 0111:B4) was purchased from Sigma-Aldrich and was dissolved in PBS. hCASMCs were treated with LPS (500 ng/ml) for 24 h following 72 h of virus transduction or for 48 h following 96 h of virus transduction.

Dexamethasone (Sigma-Aldrich) was dissolved in DMSO, and a final concentration of 3 μM was used in the experiments. hCASMCs were transduced with virus for 72 h and then treated with dexamethasone for additional 24 h before harvesting for RT-PCR. Controls received vehicle as appropriate.

### RNA Isolation and RT-qPCR

After viral transduction or treatment with agents, cells were washed in cold phosphate-buffered saline (PBS, Sigma-Aldrich, P4417) and lysed in Qiazol (Qiagen, #79306). RNA was isolated using the Qiagen miRNeasy mini kit (Qiagen, #217004) in a QIAcube workstation. To determine RNA purity and concentration we used the NanoDrop 2000c (Thermo Scientific) instrument. For RT-qPCR we used the Quantifast SYBR Green RT-PCR kit (Qiagen, 204156) and QuantiTect Primer assays from Qiagen [*CCL2* (QT00212730), *IL6* (QT00083720), *IL1B* (QT00021385), *CXCL8* (QT00000322), *SRF* (QT00084063), *ACTA2* (QT000088102), *CNN1* (QT00067718), *RELA* (QT01007370), *RELB* (QT00038640), *NFKB1* (QT00063791), *NFKB2* (QT00012404), *CEBPD* (QT00219373), *SOCS3* (QT00244580), *TGFB3* (QT00001302), *MRTFA* (QT00067921), *18S* (QT00199367), *Il1b* (QT01048355), *Srf* (QT00126378), *18s* (QT02448075)] to amplify target genes in the StepOnePlus qPCR cycler (Applied Biosystems). Qiagen considers the exact primer sequences proprietary. We used *18S* or *18s* as a housekeeping reference gene and the Pfaffl method to calculate the fold changes (vs. Null or U6).

### Confocal Imaging

Cells were fixed in 4% PFA in physiological buffer for 30 min, then permeabilized and blocked using physiological buffer with 1% BSA, 1% goat serum and 1% triton for 2 h. Cells were labeled with primary antibody overnight in the same buffer without detergent (1% BSA, 1% goat serum). The next day, cells were washed and labeled with secondary antibody for 4 h. Nuclei were stained using Hoechst at the last step. For imaging, nuclei were localized using low resolution overviews and imaged using a minimal pinhole centered at the nuclei to obtain signal within the nuclei and minimizing out of focus signal. For analysis FIJI was used. Nuclei were segmented using a threshold in the Hoechst channel and all the signal inside the nuclei was measured as mean gray value for both GFP and MRTF-A-antibody stains.

### Correlation Analyses Using GTEX Data

RNA-sequencing data from human organs was downloaded in 2020 from the GTExPortal.org (Consortium, 2013; Consortium, 2015) using R-scripts described elsewhere (Krawczyk et al., 2015; Sward et al., 2019). Transcript read counts (in TPM, transcripts per million) for *SRF*, *MYOCD*, *MRTFA*, and *MRTFB* were correlated with 13 inflammatory transcripts identified in an RNA-seq experiment where MYOCD was overexpressed. The study describing this RNA-seq experiment was submitted in parallel to the Frontiers’ theme in Cardiovascular mechanobiology (Liu et al., 2021) and has been deposited with the temporary submission ID SUB9688745, and release date 2022-06-01 (or with the release of linked data). Correlation matrices for MRTFs and the inflammatory transcripts were generated using the Pearson method using GraphPad Prism in all three arteries represented in the GTExPortal.

### THP1 Cells and Plasmid Transfection

The human THP-1 monocyte cell line was purchased from ATCC and cultured in RMPI-1640 medium supplemented with GlutaMAX (Thermo Scientific, 61870036), 10% FBS and antibiotics (penicillin 50 U/ml, streptomycin 50 μg/ml). The cells were grown in a water-jacketed cell incubator at 37°C and 5% CO_2_ in air. The medium was renewed every 2–3 day and the cells were passaged once the cell density reached 8 × 10^5^ cells/ml.

THP-1 cells were transfected with the p3xFLAG-MKL1 plasmid (Addgene, plasmid #11978) using Lipofectamine LTX Reagent with PLUS Reagent (Invitrogen, 15338030) according to the manufacturer’s instructions for 96 h before the cells were harvested and RNA isolated (miRNeasy, Qiagen).

### Protein Isolation and Western Blotting

Following virus transduction and LPS-treatment, hCASMCs were washed with cold (4°C) PBS, harvested in SDS sample buffer, and lysed by sonication on ice for 10 s. Total protein concentration was determined using the BioRad DC protein assay (BioRad, #5000112) and adjusted to ensure equal protein concentrations across samples (1 μg/μl). Protein lysates were loaded on SDS-PAGE Criterion TGX 4-15% or Any-kD precast gels (BioRad, #5671084, #5671124) and proteins were transferred to 0.2 μm nitrocellulose membranes (BioRad, #1704159) using the Trans-Blot Turbo Transfer System (BioRad). To be able to detect all the protein targets, the lysates were sometimes loaded as technical replicates. The membrane was blocked for 2 h with 1% casein/TBS (1:1) (BioRad, #1610782) in room temperature and then cut horizontally using the ladder as guidance. The membrane strips were then incubated with monoclonal primary antibodies as follows: MCP-1 (CCL2, 1 μg/ml, Abcam, ab9669), IL8 (CXCL8, 1:500, Cell Signaling, #94407), RelA (NFκB/p65, 1:1000, Cell Signaling, #8242), RelB (1:1000, Cell Signaling, #10544), NFκB1 (p105/p50, 1:1000, Cell Signaling, #13586), NFκB2 (p100/p52, 1:1000, Cell Signaling, #4882), HSP90 (1:1000, BD Biosciences, #610418), and GAPDH (1:3000, Merck Millipore, #MAB374) for 96 h at 4°C. To visualize the protein bands, membranes were incubated with HRP-conjugated secondary antibodies (1:5000, Cell Signaling, #7076 and #7074) for 2 h and the bands were detected using Supersignal West Femto substrate (Thermo Fisher Scientific, #34096) and the LI-COR Odyssey Fc instrument (LI-COR Biosciences). For quantification, all band were normalized to their respective loading controls (HSP90 and/or GAPDH) on the same membrane.

### ELISAs

Enzyme-linked immunosorbent assays (ELISAs) were performed to measure IL-8 and MCP-1 protein levels in lysates of hCASMC treated with LPS. To obtain cell lysates, cells were harvested in cold PBS and sonicated 3 × 10 s on ice. The lysate was then centrifuged at 1800 × g for 5 min at 4°C and the supernatant was collected. The assays were performed using the Human IL-8/CXCL8 DuoSet ELISA kit (#DY208) and the Human CCL2/MCP-1 Quantikine ELISA kit (#DCP00), both from R&D Systems. We adhered to protocols provided by the manufacturer.

### Co-immunoprecipitation

Co-immunoprecipitation (co-IP) of MRTF-A-binding proteins was performed using the Pierce co-IP kit (Thermo Scientific, #26149) according to the manufacturer’s instructions. Briefly, ∼26 μg purified MRTF-A antibody (Bethyl Laboratories, #A302-202A) was immobilized to the AminoLink Plus Coupling Resin in a column for 2 h in room temperature. To rule out non-specific interactions with the resin, a column containing Control Resin provided with the kit was used as a negative control. hCASMCs were washed with PBS and lysed in cold Lysis/Wash Buffer. The lysate was pre-cleared using Control Agarose Resin and 1 mg of the lysate was added to both columns and incubated overnight at 4°C. The resins were washed with IP Lysis/Wash Buffer before the MRTF-A protein complexes were eluted in Elution Buffer. The samples were analyzed by western blotting. For this, the eluted proteins were mixed with Lane Marker Sample Buffer and 100 mM DTT (Sigma-Aldrich), separated on an SDS-PAGE Criterion TGX 4–15% precast gel (Bio-Rad), transferred to a nitrocellulose membrane and blocked for 2h in room temperature. To detect MRTF-A-RelA protein interaction, the membrane was incubated for 3 days with primary RelA antibody (NFκB/p65, 1:1,000, Cell Signaling, #8242), and using MRTF-A primary antibody (MKL1/MRTF-A, 1:1,000, Cell Signaling, #14760) as a positive control. Immunoreactivity for RelA, RelB and MRTF-A was also assessed by Dot Blot. Briefly, 1 μl of the eluate was dotted onto a nitrocellulose membrane. The membrane was blocked with 1% casein/TBS (1:1) for 2 h in room temperature and then incubated for 3 days in primary antibodies for RelA (NFκB/p65, 1:1,000, Cell Signaling, #8242), RelB (1:1,000, Cell Signaling, #10544) and MRTF-A (MKL1/MRTF-A, 1:1,000, Cell Signaling, #14760). To visualize the proteins of interest for both western blot and dot blot, the membranes were incubated with HRP-conjugated secondary antibodies (1:5,000, Cell Signaling, #7076 and #7074) for 2 h and the immunoreactivity was detected using the SuperSignal West Femto substrate. Images were acquired using the LI-COR Odyssey Fc instrument (LI-COR Biosciences).

### Promoter Reporter Assay in HEK293 Cells

A plasmid containing the *CXCL8* promoter in a luciferase reporter vector was purchased from Tebu-bio (Gene information, 217HPRM30547-PG04). HEK293 cells were seeded in 24 well plates and the media were changed for antibiotic-free DMEM medium (contain 10% FBS) after 24 h. The CXCL8 promoter reporter plasmid (0.25 μg) and p3xFLAG-MKL1 plasmid (0.25 μg, Addgene, #11978) were co-transfected into HEK293 cells using Lipofectamine 2000 (Thermo Fisher Scientific, #11668030) following the manufacturer’s protocol. 96 h after transfection, medium was collected to measure the luciferase activity and the alkaline phosphatase release separately using the Secrete-Pair Dual Luminescence Assay Kit (Tebu-bio, #LFO32).

### Knockout of Srf

Inducible and SMC-specific knockout of Srf in mice was accomplished as described (accompanying paper submitted to Frontier’s theme on mechanobiology, Liu et al., 2021). Floxed Cre-negative mice treated with tamoxifen (TC) and Floxed Cre-positive mice treated with vehicle (VC) were used as controls. Mice were sacrificed on day 20 and day 21 after the first tamoxifen injection. After sacrifice, the aorta was cleaned in physiological buffer using microdissection instruments. It was then blotted on filter paper to remove excess fluid and frozen on dry ice. Ten control mice (5 VC, and 5 TC) and 12 knockout mice (TKO) were used, but one of the TC mice was excluded due to low RNA yield. RNA was isolated as described above.

## Data Availability Statement

The datasets presented in this study can be found in online repositories. The names of the repository/repositories and accession number(s) can be found below: BioSample database and accessions SAMN19277810, SAMN19277811, SAMN19277812, SAMN19277813, SAMN19277814, SAMN19277815, SAMN19277816, and SAMN19277817 (https://www.ncbi.nlm.nih.gov/biosample/19277810; https://www.ncbi.nlm.nih.gov/biosample/19277811; https://www.ncbi.nlm.nih.gov/biosample/19277812; https://www.ncbi.nlm.nih.gov/biosample/19277813; https://www.ncbi.nlm.nih.gov/biosample/19277814; https://www.ncbi.nlm.nih.gov/biosample/19277815; https://www.ncbi.nlm.nih.gov/biosample/19277816; and https://www.ncbi.nlm.nih.gov/biosample/19277817).

## Ethics Statement

The animal study was reviewed and approved by the Malmö—Lunds djurförsöksetiska nämnd, approval number 5-8-18-16388/2020.

## Author Contributions

LL, EB, CR, B-ON, and KS participated in the study design. LL and EB collected data. BM and KGS were responsible for imaging. KS generated the funding. KS wrote the manuscript, and all authors were involved in manuscript revisions. All authors have read and approved the submitted version.

## Conflict of Interest

The authors declare that the research was conducted in the absence of any commercial or financial relationships that could be construed as a potential conflict of interest.

## Publisher’s Note

All claims expressed in this article are solely those of the authors and do not necessarily represent those of their affiliated organizations, or those of the publisher, the editors and the reviewers. Any product that may be evaluated in this article, or claim that may be made by its manufacturer, is not guaranteed or endorsed by the publisher.
